# Modulation of Immune Response to *Chlamydia muridarum* by Host miR-135a

**DOI:** 10.3389/fcimb.2021.638058

**Published:** 2021-04-13

**Authors:** Jonathon Keck, James P. Chambers, Jieh-Juen Yu, Xingguo Cheng, Lane K. Christenson, M. N. Guentzel, Rishein Gupta, Bernard P. Arulanandam

**Affiliations:** ^1^ South Texas Center for Emerging Infectious Diseases, Department of Biology, University of Texas at San Antonio, San Antonio, TX, United States; ^2^ Department of Materials & Bioengineering, Southwest Research Institute, San Antonio, TX, United States; ^3^ Department of Molecular and Integrative Physiology, University of Kansas Medical Center, Kansas City, KS, United States

**Keywords:** *Chlamydia*, microRNA, miR135a, cell migration, dendritic cells

## Abstract

Previously, our laboratory established the role of small, noncoding RNA species*, i.e.*, microRNA (miRNA) including miR-135a in anti-chlamydial immunity in infected hosts. We report here chlamydial infection results in decreased miR-135a expression in mouse genital tissue and a fibroblast cell line. Several chemokine and chemokine receptor genes (including CXCL10, CCR5) associated with chlamydial pathogenesis were identified *in silico* to contain putative miR-135a binding sequence(s) in the 3’ untranslated region. The role of miR-135a in the host immune response was investigated using exogenous miR-135a mimic to restore the immune phenotype associated with decreased miR-135a following *Chlamydia muridarum* (Cm) infection. We observed miR-135a regulation of Cm-primed bone marrow derived dendritic cells (BMDC) *via* activation of Cm-immune CD4^+^ T cells for clonal expansion and CCR5 expression. Using a transwell cell migration assay, we explore the role of miR-135a in regulation of genital tract CXCL10 expression and recruitment of CXCR3^+^ CD4^+^ T cells *via* the CXCL10/CXCR3 axis. Collectively, data reported here support miR-135a affecting multiple cellular processes in response to chlamydial infection.

## Introduction

*Chlamydia trachomatis* (Ct) is the leading cause of bacterial sexually transmitted infection (STI), and preventable blindness worldwide. In the U.S. alone, over 1.8 million cases were reported in 2018 (CDC, 2019). An accurate number of infected individuals is not known due to the high level of asymptomatic infections in men and women*, i.e.*, ~50 and 75%, respectively (Darville and Hiltke, 2010). If left untreated in symptomatic and asymptomatic women, Ct induces pathology leading to a whole host of reproductive sequelae including cervicitis, pelvic inflammatory disease (PID), ectopic pregnancy, and infertility (Darville and Hiltke, 2010). The lack of organized healthcare programs in several developing or under-developed countries (Lanjouw et al., 2015), absence of a licensed vaccine (De la Maza et al., 2017), and compounding risk factors (Singh and Marrazzo, 2013; Rey-Ladino et al., 2014; Falasinnu et al., 2015) have collectively resulted in the rising incidence of Ct globally (Yu et al., 2016).

Interest in the effect(s) of non-coding RNA on control of immune signaling has increased. MicroRNA (miRNA/miRs) has been shown to play a significant role in innate and adaptive immune signaling in response to invading bacteria (Keck et al., 2017). The influence of miRs in optimizing the immune response is analogous to other intracellular regulatory events, *e*.*g*., phosphorylation and histone modification. Typically, miRs modulate gene function *post* transcriptionally by binding to target mRNAs leading to increased mRNA degradation with concomitant reduction of translation (Bartel, 2004).

The role of specific miRs in genital Ct infection has been established by our group as well as others (Arkatkar et al., 2015; Gupta et al., 2015; Gupta et al., 2016; Yeruva et al., 2017). Several reports suggest miR-135a may play an important role in proliferation and migration of immune and non-immune cells within the inflammatory *milieu* (Navarro et al., 2009; Wan et al., 2016; Zeng et al., 2016). In this study, we sought to examine involvement of miR-135a in chlamydial infection using the murine *Chlamydia muridarum* (Cm) infection model. Identification of host response immune regulator molecules may lead to new and effective treatment strategies for this leading global cause of bacterial STIs.

## Methods

### *Chlamydia muridarum*

Cm seed stocks were propagated in HeLa 229 cells. At 24 hours *post* infection, HeLa cells were mechanically disrupted, and following centrifugation, bacterial pellets were purified on a Renografin (E.R.Squibb and Sons, *Inc*., Princeton, NJ, USA) gradient as previously described (Gupta et al., 2016). The same Cm seed stock was used throughout this study.

### Intravaginal Challenge

All experiments utilizing animals were performed in accordance with the Animal Welfare Act, the Guide for the Care and Use of Laboratory Animals of the National Institutes of Health, and approved guidelines and protocols set forth by the University of Texas at San Antonio Institutional Animal Care and Use Committee (IACUC, approved experimental protocol document MU012). Animals were euthanized by CO_2_ inhalation followed by cervical dislocation when the experimental endpoints were reached. Female (4-6 week old) wild type (WT) C57BL/6 mice (Jackson Laboratory, Bar Harbor, ME, USA) were inoculated with 5 × 10^4^ inclusion forming units (IFUs) of live Cm elementary bodies (EBs) suspended in 40 μl sucrose-phosphate-glutamic acid (SPG) buffer by intravaginal route 6 days *post* depo-progesterone administration as previously described (Gupta et al., 2015). Mice challenged with SPG alone were used as mock control.

### Genital Tract Isolation

At the indicated time following infection, mice were euthanized, and genital tract tissue (cervix, uterine horns, and oviducts) was removed aseptically for either immediate cell isolation or storage of tissues at -80°C for downstream RNA isolation and analysis.

### Genital Tract Cell Isolation

Genital tract tissue was minced, transferred to 5 mL culture media containing collagenase (500 U/mL), and vigorously stirred for 1 hour to dissociate tissue as previously described (Jiang and Kelly, 2012). The resulting heterogenous tissue cell suspension (comprised primarily of mucosal epithelial cells, tissue endothelial cells, and immune cells such as macrophages, dendritic, and CD4^+^ T cells) was passed through a 100 µm cell strainer, and cells were harvested by centrifugation (200 x g for 5 minutes). Bone marrow derived dendritic cells (BMDC) and splenic CD4^+^T cells were prepared as previously described (Gupta et al., 2016).

### Genital Tract Tissue Powder Preparation

Genital tract tissue was snap-frozen, pulverized using a mortar and pestle, and the resulting genital tract tissue frozen powder served as source of total RNA (Gupta et al., 2015).

### Flow Cytometry

Cells were suspended in Fc Block (BD Biosciences, San Jose, CA, USA) and PBS containing 0.5% (v/v) FBS for 15 minutes. Following incubation, cells were surface stained with mouse specific antibodies (BD Biosciences); anti-mouse: CD3 (BV605), CD4 (perCP Cy5.5), and CXCR3 (PE) for 30 minutes. Cell suspensions were washed twice, resuspended in PBS containing 0.5% (v/v) FBS and FACS solution, and phenotype determination assessed using an LSR II instrument, and FACSDiva software (BD Biosciences).

### Quantitative Reverse Transcriptase (qRT)-PCR

Total RNA was obtained from genital tissue powder, cell lines, and murine primary cells using either a miRNeasy RNA extraction Kit (Qiagen, Hilden, DE, USA) or Aurum Total RNA Fatty and Fibrous Tissue Kit (Bio-Rad Laboratories, Hercules, CA, USA) according to manufacturer’s instructions. Extracted RNA was assessed using a Nanodrop Spectrophotometer (ThermoScientific, Asheville, NC). RNA samples (1 µg with A_260/280_ and A_260/230_ values of ≅ 2.0 and 1.8 or higher, respectively) were converted to cDNA.

RNA was converted to cDNA using a miScript-II-RT (Qiagen) or iScript Advanced cDNA Synthesis Kit for RT-qPCR according to manufacturer’s recommendations. Amplification was carried out using the Bio-Rad PrimePCR™ SYBR Green Assay (BioRad Laboratories, Hercules, CA, USA), with proprietary forward and reverse primers for mouse CXCL10 (qMmuCED0001068), CXCL12 (qMmuCID0019961), and CCR5 (qMmuCED0051515). MiR-135a expression was determined using the Qiagen hsa-miR-135a-5p miRCURY LNA miRNA (SYBR Green) PCR Assay. Amplification was carried out using a BioRad CFX96 Touch Real-Time PCR Detection System (Bio-Rad Laboratories). Quantitation-normalization of gene expression was achieved using BioRad CFX Maestro software (Bio-Rad Laboratories). MiRNA expression was normalized using Snord68 small nucleolar RNA (Qiagen) which has been shown to exhibit stable expression across different tissues and cell types; whereas, Gapdh and HSP90 housekeeping genes were used for determination of expression of all other gene targets. Differences in miR and gene expression with respect to respective control groups (mock infected, mock treated, and media alone) were determined using the 2^(−Average ΔΔCq)^ method of Livak and Schmittgen (2001).

### *In Vitro* miRNA Transfection

Transfection of cells with MiR-135a mimic (5’-UAUGGCUUUUUAUUCCUAUGUGA-3’, Qiagen) was conducted using 20 µM Attractene transfection reagent per manufacturer’s (Qiagen) recommendations. Using this established protocol, transfection efficiencies >80% (data not shown) have been previously reported by this laboratory (Gupta et al., 2015). Transfection was carried out for 18-24 hours prior to downstream applications.

### Cell Proliferation

BMDC CD11c^+^ and splenic CD4^+^ T cells were enriched using cell specific isolation kits per manufacturer’s instructions (Stemcell Technologies, Vancouver, WA, USA). Purified CD11c^+^ cells were plated at 2 x 10^5^ cells per well, treated with media alone or mmu-miR-135a-mimic for 24 hours, and subsequently infected with Cm (MOI = 1) for 24 hours. Enriched CD4^+^ T cells from SPG (mock) or Cm infected mice (12 days *post* infection) were stained with BD Horizon™ Violet Proliferation Dye 450 (VPD, BD Biosciences, San Jose, CA, USA) for 30 minutes followed by co-culturing with BMDCs for 72 hours. Following cell division, the VPD450 dye is distributed uniformly amongst daughter cells with each daughter cell retaining approximately one-half of the parental cell VPD450 fluorescence intensity. The number of VPD450 positive CD4^+^ T cells and fluorescence intensity were determined by flow cytometry and % T cell proliferation calculated.

### Cell Migration

Cell migration was assessed as previously described (Gomez-Lopez et al., 2011) using a modified Boyden Chamber. Genital tract tissue from Day 6 mock infected or Cm infected C57BL/6 mice was disassociated (*cf*., tissue isolation), and cells were seeded at 5 x 10^5^ per well. Cells were transfected with mmu-miR-135a mimic for 24 hours or left untreated. Splenic CD4^+^ T cells were isolated from Day 6 mock and Cm infected C57BL/6 mice using an EasySep Mouse CD4^+^ T Cell Isolation Kit per manufacturer’s instructions (Stemcell Technologies, Vancouver, WA, USA). The upper well of the Boyden Chamber containing a 3 μm screen (Corning, NY, USA) was seeded with 5 x 10^5^ cells. An upper insert containing isolated splenic CD4^+^ T cells or no cells (as control) was introduced into respective wells containing genital tract cells, and incubated for 14 hours. Lower well contents were collected, and subjected to flow cytometry. The number of CD4^+^ CXCR3^+^ T cells that migrated from the upper chamber was determined by subtraction of the number of CD4^+^ CXCR3^+^ T cells obtained from the respective ‘no cell’ control. The contents of the lower well were used for determination of *1)* gene expression by qRT-PCR and *2)* cytokine (IFN-γ) production by ELISA (BD OptEIA Mouse IFN-γ ELISA Set).

### *In Silico* Analysis

Bioinformatic analysis for putative related miR binding sites in genes encoding proteins observed to be modulated by *C. muridarum* infection was accomplished using a miR target predictive algorithm (www.microRNA.org, Memorial Sloan-Kettering Cancer Center, NY). GraphPad Prism 5 (La Jolla, CA) was used to perform all tests of significance. The *in silico* predicted Mir-135a regulated gene sequences are listed in **Table I**.

**Table 1:** *In silico* predicted Mir-135a regulated gene sequences.

Gene	Category	Sequence*
**CCR5**	Receptor	uguugugcccc**U**C**A**G**AAGCCAU**g
**CXCL10**	Ligand	uccugc**AGGA**UG**AU**GGUC**AAGCCAU**g
**CXCL12**	Ligand	ugacaguuauu**U**G**A**G**AAGCCAU**u

*Bold capital letters represent predicted miR-135a binding sequences.

## Results

### MiR-135a Levels Are Decreased in Response to *C. muridarum*

**Figure 1:**
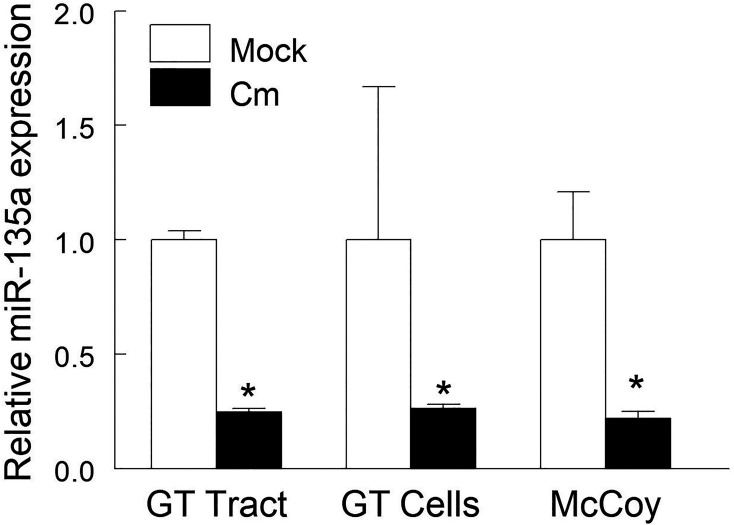
*Chlamydia muridarum* infection down-regulates miR-135a. C57BL/6 mice (n = 3) were intravaginally challenged with Cm (5 × 10^4^ IFUs in SPG buffer) or SPG buffer alone (mock). Genital tract tissue was collected at day 6 *post*-challenge and miR-135a expression was measured. Cell suspensions of naïve mouse genital tract and fibroblast cell line (McCoy) cells were exposed to Cm (2 x 10^5^ IFUs, MOI = 1) for 24 hours in a 24-well microplate. Relative expression of miR135a in Cm infected tissue/cells compared to the respective uninfected tissue/cells was determined by qRT-PCR and calculated using the 2−ΔΔCT method. Data shown are representative of 3 experiments. *p < 0.05 Student t test.

Using an 88 immuno-pathologic miR array, we previously identified 11 miRs (including miR-135a) that were modulated in the genital tract *post* chlamydial challenge (Gupta et al., 2015). In order to ascertain the role of miR-135a in *Chlamydia* pathogenesis, we first confirmed that its expression is regulated by Cm infection. qRT-PCR quantification of miR-135a expression in genital tract tissue revealed a 75% decrease at day 6 *post* intravaginal Cm challenge (**Figure 1**). Furthermore, *in vitro* chlamydial infection (24 hours) of genital tract single-cell suspensions and McCoy cells (a murine fibroblast cell line commonly employed to propagate chlamydial strains) resulted in a 76 and 78% reduction of miR-135a expression, respectively (**Figure 1**). These results suggest Cm infection down-regulates miR-135a. Subsequent *in silico* analysis of potential miR-135a target genes resulted in identification of several chemokine and chemokine receptor genes including CCR5, CXCL10 and CXCL12 containing putative miR-135a binding sequences in their respective 3’ untranslated region (**Table 1**). Host response down-regulation of miR-135a following Cm infection may prevent degradation of these gene transcripts thus allowing disease resolution. In order to assess restoration of decreased miR-135a following Cm infection, an *in vitro* approach using primary cells isolated from mock and Cm infected mice with and without administered miR-135a mimic was employed.

### miR-135a Plays a Role in Dendritic Cell Mediated Chlamydial Antigen-Specific CD4^+^ T Cell Activation

**Figure 2:**
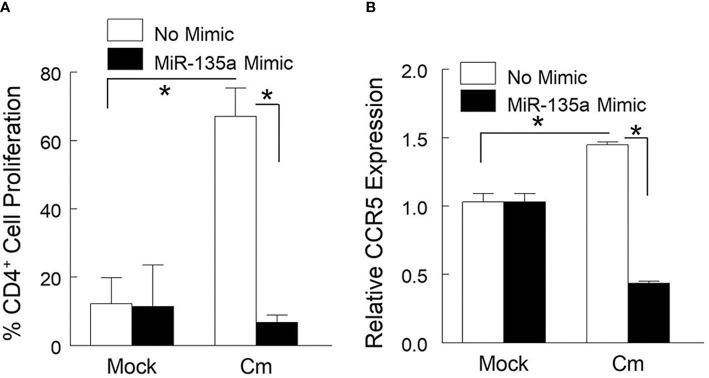
miR-135a affects dendritic cell mediated Cm-primed CD4^+^ T cell proliferation and CCR5 gene expression. Bone marrow derived dendritic cells (BMDC) were stimulated with media alone, or media containing miR-135a mimic for 24 hours followed by exposure to Cm (MOI = 1). Cm primed BMDCs were co-cultured with splenic CD4^+^ T cells from day 6-*post* intravaginally challenged mice (5 x 10^4^ IFU) or mock infected. **(A)** Effect of miR-135a mimic on CD4^+^ T cell proliferation. **(B)** Effect of miR-135a mimic on CCR5 gene expression. Data shown are representative of 3 experiments. *p < 0.05 Student t test.

Dendritic and Th1 cells are important for immune defense against genital chlamydial infection. It has been reported that CCR5 expressing CD4^+^ T cells are critical for host protection against chlamydial infection (Belay et al., 2002; Barr et al., 2005; Olive et al., 2011). Therefore, we assessed in BMDC the contribution of miR-135a in T cell proliferation and CCR5 gene expression by co-culturing Cm-exposed BMDC with CD4^+^ T cells from Cm infected mice. Specifically, BMDC prepared from naïve mice and transfected ormock-transfected with miR-135a mimic were exposed to Cm for 24 hours followed by co-culturing with CD4^+^ T cells enriched from Cm infected or uninfected (mock) spleen. As shown in **Figure 2A**, Cm-exposed naïve BMDCs with or without miR135a mimic transfection minimally activate naive CD4^+^ T cells for proliferation (less than 12%). In contrast, 67.07% ± 4.13 of Cm-primed CD4^+^ T cell population underwent proliferation following activation by Cm-primed BMDCs. This antigen specific BMDC-mediated T cell proliferation was abrogated when BMDCs were transfected with miR135a mimic (10.60% ± 3.77, p<0.05). Furthermore, CCR5 gene expression in naïve CD4^+^ T cells was not altered by incubation with Cm-primed BMDCs with or without transfected miR-135a mimic (**Figure 2B**). In contrast, Cm-exposed BMDC activated Cm-primed T cells indicated a 1.44 ± 0.01fold-increase in CCR5 gene expression in the absence of miR-135a mimic but a 56% decrease in the presence of miR-135a mimic compared to the corresponding naïve (mock) T cell control (**Figure 2B**).

### miR-135a Plays a Role in Immune T Cells Migration to Genital Track Following Chlamydial Infection

**Figure 3:**
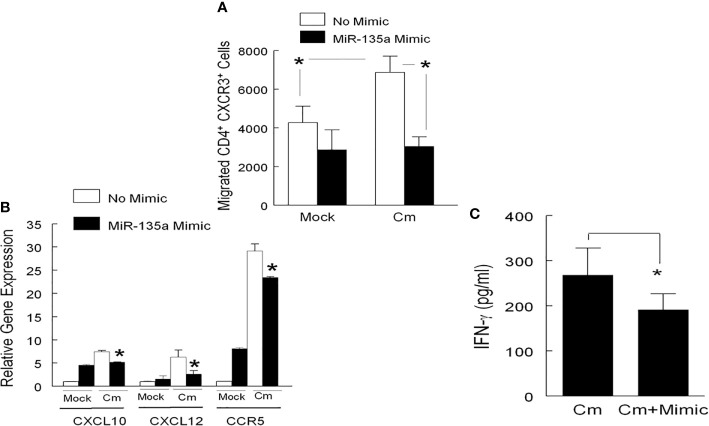
miR-135a affects CD4^+^ T cell migration. T cell migration was determined using a modified 24-well Boyden Chamber. Genital tract tissue cell suspensions from mock or Cm infected (day 6) mice were added to the lower chamber (5 x 10^5^ per well), and transfected with miR-135a mimic or media alone for 24 hours prior to determination of migration. Enriched CD4^+^ T cells (from day 6 Cm infected splenocytes) were added to the upper chamber (5 x 10^5^ cell per well). Fourteen hours *post* addition of cells to the upper chamber, the lower chamber contents were used for **(A)** enumeration of migrated CXCR3-expressing CD4^+^ T cells by Flow cytometry, **(B)** comparison of relative CXCL10, CXCL12, and CCR5 gene expression in Cm infected cells transfected with and without miR-135a mimic to the uninfected/non-transfected control group (expression level normalized to 1), and **(C)** determination of IFN-γ secretion. Data shown are representative of 3 experiments. *p < 0.05 Student t test.

In primary Cm infection, the initial immune response in the mouse genital tract is dominated by myeloid cell infiltrates, including neutrophils, followed by recruitment of T cells (CD4^+^ T cells in particular) during the resolution stage (Morrison and Morrison, 2000). Considering the presence of a predictive miR-135a binding target sequence in the 3’ UTR of chemokine CXCL10 mRNA **(** **Table 1** **)**, we hypothesized that miR-135a could disrupt CD4^+^ T cell migration through the CXCL10/CXCR3 axis. To test this hypothesis, we measured migration of CXCR3^+^ CD4^+^ T cells to CXCL10 secreting genital cells using a combined Boyden chamber-Flow Cytometry Assay. Specifically, mouse genital tract cells isolated from day 6 mock and Cm infected animals were seeded in the lower well of Boyden chamber, and either transfected with miR-135a mimic or left untreated. Splenic CD4^+^ T cells isolated from Day 6 Cm infected mice were placed in the upper well of the Boyden chamber. As shown in **Figure 3A** using a gating strategy **(** **Supplementary Figure 1** **)**, migration of splenic CD3^+^CD4^+^ T cells expressing the CXCR3 marker increased approximately 61% after incubation with Cm-infected genital cells (6873 ± 839) compared to incubation with uninfected cells (4269 ± 854). Transfection of Cm-infected genital tract cells with miR-135a mimic significantly reduced CXCR3^+^ CD4^+^ T cell migration approximately 56%, *i.e.*, from 6873 ± 839 to 3047 ± 493 cells. Furthermore, we analyzed the total cell population present in lower wells following T cell migration (14 hours) for chemotaxis gene expression and effector cytokine secretion. As shown in **Figure 3B**, Cm-infected genital tract cells interacted with Cm-primed CD4^+^ T cells resulting in increased CXCL10 gene expression (7.49 ± 0.11) compared to the uninfected cell control (normalized as 1). The observed increase in CXCL10 gene expression was reduced in Cm-infected genital tract cells transfected with miR-135a mimic (from 7.49 ± 0.11 to 5.18 ± 0.04-fold) which correlated with reduction of CXCR3^+^ CD4^+^ T cell migration (**Figure 3A**). Other Cm infection induced cell migration/homing immune molecules were also affected by miR135-a mimic transfection including reduced CXCL12 (6.33 ± 0.84 to 2.65 ± 0.42), and CCR5 (29.13 ± 0.89 to 23.43 ± 0.11) (**Figure 3B**). Effector Th1 T cells producing INF-γ have been shown to be critical in resolving genital tract Cm infection (Perry et al., 1997; Morrison and Caldwell, 2002; Li et al., 2008). Interaction of genital tract cells collected from Cm infected mice with CD4^+^ T cells induced IFN-γ production secretion (268 ± 61 pg/ml, *p* < 0.05), and transfection of these Cm-infected genital tract cells with miR135a mimic significantly reduced IFN-γ production (192 ± 35 pg/ml) which may partially be due to the reduced CD4^+^ T cell migration to the lower chamber (**Figure 3C**).

## Discussion

The miR-135a gene (mir135a-2) is located on chromosome 10 (Chr10:92072086-92072185 bp, - strand), and is known for its role as an oncogenic miRNA which controls tumor aggressiveness in several cancers including colon, melanoma, breast, and prostate (Wu et al., 2012; Zhou et al., 2012; Yamada et al., 2013; Kroiss et al., 2015; Zeng et al., 2016; Fukagawa et al., 2017). The regulatory role of miR-135a in cancer progression by either promoting or suppressing tumor cell growth/migration has been well documented (Wu et al., 2012; Yamada et al., 2013; Zeng et al., 2016; Xie et al., 2019; Zhang et al., 2019); however, its role in infectious diseases is largely unknown.

The proliferative responses observed in inflamed microenvironments has been reported to be controlled by *FOXO1* in a miR-135a dependent fashion (Mao et al., 2015; Ren et al., 2015; Zeng et al., 2016). Importantly, because of the putative regulation of transcription factor *STAT-6* (Pandey et al., 2016), or proliferation factors such as *ROCK1, HOXA10, and BCL-2*, the cellular therapeutic potential of miR-135a may hold promise (Yang and Yin, 2017). To this end, the *in vivo* regulation of *GATA3*, a cellular Th_2_ lineage marker for miR-135a has been used in correcting the Th_1_/Th_2_ imbalance in acute allergic rhinitis (Luo et al., 2014), and is thus proposed as a therapeutic agent for intervention of mast cell and allergen induced inflammation (Deng et al., 2015).

Pursuing our previous findings (Arkatkar et al., 2015; Gupta et al., 2015; Keck et al., 2017), we report here an unrecognized but suggested role for miR-135a, *i.e*., regulation of immune gene expression following chlamydial infection. Intravaginal (*i. vag*) infection of mice by Cm significantly reduced overall miR-135a expression in genital tract tissue. This Cm-mediated reduction of miR-135a was demonstrated *in vitro* using genital tract tissue cell preparations and a murine fibroblast cell line. Although absent bacterial burden data needed to conclusively show increasing disease severity in the presence of a miR-135a mimic countering the suggested Cm-induced down-regulation of miR-135a, we do demonstrate the immune-regulatory role of miR-135a as evidenced by alteration of effector T cell activation and infection site homing. Specifically, we observed that miR-135a can regulate 1) CCR5 expression and cell proliferation of Cm-primed CD4^+^ T cells when activated by Cm-exposed BMDC, and 2) migration of Cm-primed CD4^+^ T cells to Cm-infected genital tract cells *via* CXCL10/CXCR3 axis mediated immune activation. These miR-135a mediated effects on immune cell proliferation and/or migration are consistent with previously implicated chemokine and chemokine receptor function in Ct/Cm infection (Belay et al., 2002; Olive et al., 2011). The ability of miR-135a to disrupt CD4^+^ T cell proliferation in concert with expression of CCR5 (**Figures 2** and **3**) is suggestive of its potentially important role in immune cell trafficking following infection. Additionally, in the presence of miR-135a mimic, a decrease in the migratory index of splenic CD4^+^ CXCR3^+^ T cells were observed (**Figure 3** and **Supplementary Figure 1**). Expression of CXCR3 in CD4^+^ T cells has been shown to alter migration patterns in *Chlamydia* infected hosts (Olive et al., 2011). This observation is congruent with that of others defining the important role for CXCL10 in migrating T cells (Bonecchi et al., 1998; Dufour et al., 2002).

We observed miR-135a regulatory binding sites for genes previously implicated in anti-chlamydial immunity (**Table 1**) and regulation of IFN-γ (**Figure 3C**). Based on signaling axes in which these genes are involved and additional, ongoing preliminary investigations including *in silico* and wet laboratory analyses (data not shown), we hypothesize that miR-135a modulates multiple signaling pathways using the following mechanisms: (1) Type-1 and Type-2 IFN signaling through molecules such as Jak1 an IFNαR2, IL-33, CXCL10, and/or (2) alteration of canonical (MyD88) and non-canonical (Tram) signaling pathways, thereby collectively contributing to regulation of anti-chlamydial immunity via interferon signaling.  Thus, additional gain and loss of function studies are warranted to investigate these pathways.

Using our previously established dendritic effector cell: co-culture model (Gupta et al., 2016), and a modified Boyden Chamber assay (Gomez-Lopez et al., 2011), we determined the effects of miR-135a on cell proliferation (cognate interaction between dendritic cells and antigen specific CD4^+^ T cells), and cell migration in antigen specific CD4^+^ T cells, respectively (**Figures 2** and **3**). Focusing on the initial stage of *i.vag.* infection (day 6 *post* infection) (Gupta et al., 2015; Gupta et al., 2016), we interrogated the effect of miR-135a on CD4^+^ T cell function (**Figures 2** and **3**) which has been implicated in early stages of genital chlamydial infection (Maxion et al., 2004; Roan et al., 2006; Jayarapu et al., 2009; Farris et al., 2010; Lee et al., 2010; Yu et al., 2010; Olive et al., 2011; Jiang et al., 2017; Naglak et al., 2017; Lijek et al., 2018).

While our findings suggest changes at the mRNA level consistent with miR regulation of gene expression, we cannot rule out possible miR-135a independent events, *e.g*., the presence of mRNA variants, post-transcriptional silencing, and/or compensatory effects by other immune cell populations. Furthermore, we assume that the differences in immune gene function following Cm infection is at minimum antigen-based, and not Cm antigen specific. The lack of a specific cell type *in vivo* model, *i.e*., conditional knockout, fate-mapped or inducible mouse model (Mak et al., 2001; Poltorak and Schraml, 2015; Shi et al., 2018) necessitated use of differing cell lines/cell types which may exhibit differential immune presentation. Nevertheless, this is the first report of immune regulatory mechanisms in which the host response to chlamydial infection occurs *via* miR-135a. Although data reported here are primarily *in vitro* derived, they can serve as a starting point for *in vivo* study into the role of miR-135a in chlamydial pathogenesis either by intraperitoneal injection of liposome encapsulated miR-135a mimic or use of miR-135a gene knockout mice when such animals become available.

## Data Availability Statement

The original contributions presented in the study are included in the article/**Supplementary Material**. Further inquiries can be directed to the corresponding author.

## Ethics Statement

The animal study was reviewed and approved by The University of Texas at San Antonio Institutional Animal Care and Use Committee (IACUC).

## Author Contributions

JPC, MNG, RG, and BPA conceived and designed the experiments. JK carried out the experiments. XC provided reagents for experiments. JK, J-JY, MNG, JPC, RG, and BPA analyzed the data. JK, J-JY, JPC, LC, RG, and BPA wrote and edited the manuscript. All authors contributed to the article and approved the submitted version.

## Funding

This work was supported by the National Institutes of Health (NIH) grant IR03AI11771401A1, and the Army Research Office (Department of Defense contract No. W911NF-11-1-0136).

## Conflict of Interest

The authors declare that the research was conducted in the absence of any commercial or financial relationships that could be construed as a potential conflict of interest.
