# Supplementary material for: Modulation of Immune Response to Chlamydia muridarum by Host miR-135a
Source: Front Cell Infect Microbiol. 2021 Apr 13;11:638058. doi: 10.3389/fcimb.2021.638058 (PMC8076868; doi:10.3389/fcimb.2021.638058)
Supplement: Supplementary file 1 [file Image_1.pdf]

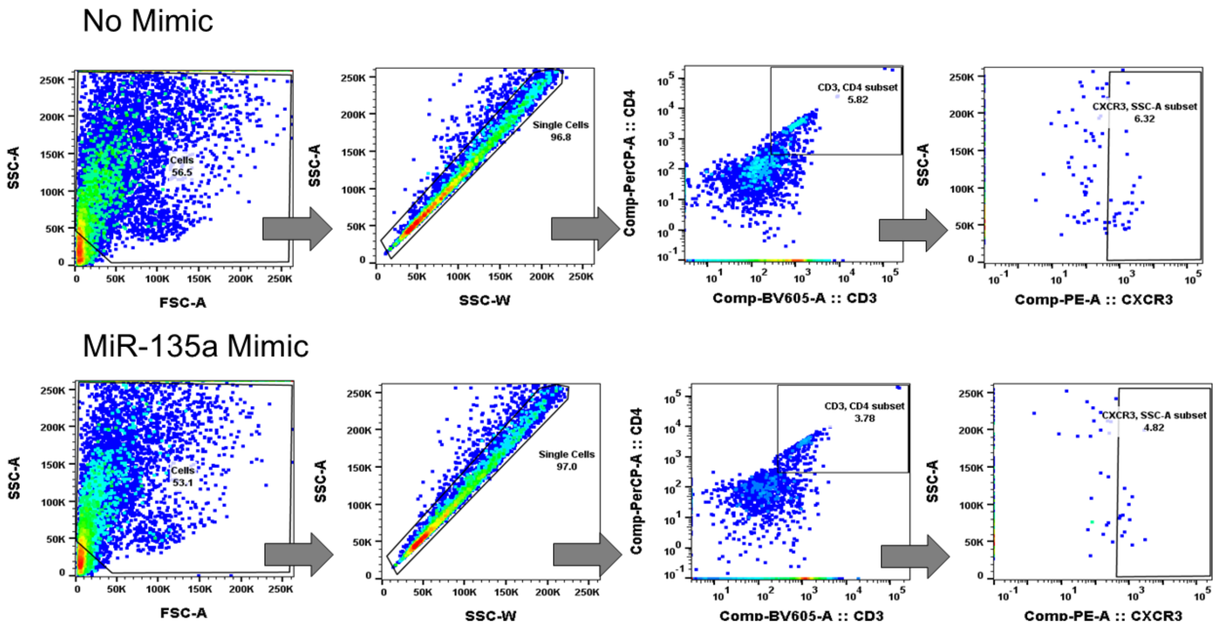


**Supplementary Figure 1. Representative Gating Strategy and Flow Cytometry Markers Used.** T cell migration was determined using a modified 24-well Boyden Chamber. Genital tract cell suspensions from Cm infected (day 6) mice were added to the lower chamber (5 x $10^5$ per well), and either transfected with miR-135a mimic or un-transfected with media alone for 24 hours prior to determination of migration. $CD4^+$ T cells enriched from day 6 Cm infected splenocytes were added to the upper chamber (5 x $10^5$ cell per well). Fourteen hours *post* addition of cells to the upper chamber, the lower chamber contents were used for enumeration of migrated CXCR3-expressing $CD4^+$ T cells by Flow cytometry. Data shown here are representative gating plots of flow cytometry markers for $CD4^+CXCR3^+$ T cell populations.
